# Funding Influencing Practice and Outcomes in Reablement

**DOI:** 10.1093/geroni/igab046.1615

**Published:** 2021-12-17

**Authors:** Matthew Parsons, Paul Rouse

**Affiliations:** 1 University of Waikato, Hamilton, Waikato, New Zealand; 2 University of Auckland, Auckland, Auckland, New Zealand

## Abstract

Internationally, Home Care is invariably funded through fee-per-service, e.g., if an hour of care is delivered, the provider receives an associated amount of funding. However, the funding model discourages reductions in packages-of-care when a client’s functional capacity improves, and further disincentivises providers to discharge clients. Similarly, staff income is often directly associated to the delivered hours-of-care and if a client’s hours are reduced, so is their income; again, discouraging the right behaviour, such as reporting improvements in independence levels. In 2008 in New Zealand, we developed a case-mix funding methodology and have been progressively implementing the new model since that time. This presentation highlights the findings in relation to how Home Care service hour allocations titrate against needs following implementation of the model as well as a number of other key quality outcomes that have been observed as a result of the case-mix model.

